# New insights of phenolic compounds from optimized fruit extract of *Ficus auriculata*

**DOI:** 10.1038/s41598-021-91913-w

**Published:** 2021-06-14

**Authors:** M. Shahinuzzaman, Parul Akhtar, N. Amin, Yunus Ahmed, Farah Hannan Anuar, H. Misran, Md. Akhtaruzzaman

**Affiliations:** 1 Department of Chemical Sciences, Faculty of Science and Technology, Universiti Kebangsaaan Malaysia, 43600 Bangi, Selangor Malaysia; 2 Department of Chemistry, Chittagong University of Engineering & Technology, Chittagong, 4349 Bangladesh; 3 Institute of Sustainable Energy, Universiti Tenaga Nasional (@The National Energy University), Jalan IKRAM-UNITEN, 43000 Kajang, Selangor Malaysia; 4 Solar Energy Research Institute, Universiti Kebangsaan Malaysia, 43600 Bangi, Selangor Malaysia

**Keywords:** Chemical biology, Drug discovery, Plant sciences, Energy science and technology

## Abstract

In this study, the extraction conditions extracted maximize amounts of phenolic and bioactive compounds from the fruit extract of *Ficus auriculata* by using optimized response surface methodology. The antioxidant capacity was evaluated through the assay of radical scavenging ability on DPPH and ABTS as well as reducing power assays on total phenolic content (TPC). For the extraction purpose, the ultrasonic assisted extraction technique was employed. A second-order polynomial model satisfactorily fitted to the experimental findings concerning antioxidant activity (R^2^ = 0.968, *P* < 0.0001) and total phenolic content (R^2^ = 0.961, *P* < 0.0001), indicating a significant correlation between the experimental and expected value. The highest DPPH radical scavenging activity was achieved 85.20 ± 0.96% at the optimum extraction parameters of 52.5% ethanol (v/v), 40.0 °C temperature, and 22 min extraction time. Alternatively, the highest yield of total phenolic content was found 31.65 ± 0.94 mg GAE/g DF at the optimum extraction conditions. From the LC–ESI–MS profiling of the optimized extract, 18 bioactive compounds were tentatively identified, which may regulate the antioxidant activity of fruits of *F. auriculata*.

## Introduction

The human body is vulnerable to reactive oxygen species (ROS). Natural antioxidants are an essential compound for reducing the concentration of these species and prevent various chronic disorders like cancer, rheumatoid arthritis, atherosclerosis, emphysema, cirrhosis, diabetes and others, which cause free radical (˙OH, ^1^O_2_, O_2_˙^−^) and non-free radical (R–OOH, NO, ONOO^−^, and H_2_O_2_) ROS species^1,2^. Besides the body’s endogenous antioxidant defence, antioxidants are primarily derived from diet and can promote good health. Numerous synthetic antioxidants are commonly used in different food products, but these products are restricted due to their carcinogenic and other toxic properties^3^. In addition, there is a demand for natural antioxidants as food preservatives to reduce oxidation and rancidity of foods. Therefore, the attention of natural antioxidants has been raised considerably in the study of certain fruits, vegetables and leaves with high antioxidant contents to boost their consumption. Consequently, an effective extraction technique and the optimization of the extraction conditions are very important for the isolation of antioxidant phenolic compounds. It may enable to obtain natural antioxidants in larger quantities and reduce costs.

*Ficus auriculata* Lour., a member of the Moraceae family, is a naturally grown plant in lowland tropical rainforests, along streams or on rocks. It is also known as Elephant ear fig or Roxburgh fig^4^. Its crude extract exhibited antioxidant, antibacterial, antimicrobial, antihyperlipidemic, hepatoprotective activity as well as contain a higher amount of flavonoid content^5–7^. Fruits of *F. auriculata* are not only valuable for its nutritional value but also contains a higher amount of phenolic compounds as compared to other parts. The previous study found that the leaves, barks, and fruits of *F. auriculata* exhibited good result with the inhibition of DPPH and ABTS scavenging activity using ultrasonic assisted extraction process^8^. Moreover, most of the extracts from *F. auriculata* obtained by ultrasound assisted extraction (UAE) process, showed the highest antioxidant activity, phenolic contents and extraction yields as compared to the maceration process^8^. In 2014, Hlail and co-workers reported similar phenomena for the fruits to extract, which exhibited higher biological activity compared to leaves extract^9^.

Numerous extraction techniques have been evolved and used to isolate the bioactive antioxidant compounds from plant sources. Among these techniques, maceration extraction^3,10^, microwave-assisted extraction^11,12^ and supercritical fluid extractions^13,14^ are now used. In the first case, it is time wasting and requires relatively large amounts of solvents. The supercritical fluid extraction process is not economically viable due to the higher cost of the equipment and blockage the systems due to the use of water as the solvent. By considering the concept of “green chemistry”, environment-friendly techniques are required for the determination of antioxidant compounds. Ultrasonic assisted extraction (UAE) is an eco-friendly method, which offers high extraction efficiency, good reproducibility in lower extraction times and requires relatively low solvent, temperature, and energy input. This method can be easily scaled up for industrial applications^15,16^.

In general, process optimization could be achieved through either statistical or experimental method^17,18^. The experiential technique involves the study of one-factor-at-a-time which is that all the variables are kept at constant and only one variable changes^19^. It also increases the experimental run to conduct the research that is laborious, time-consuming and raise the solvent and materials consumption^20^. So, it is needed to establish the optimum process to recover the highest numbers of bioactive compounds with conserved all the functional parameters. Among the various Response surface methodology (RSM) designs, Central composite design (CCD) is an efficient system which is timesaving and more competent among others. It is very much helpful to develop, improve and optimize extraction conditions of natural antioxidants and plant metabolites^21,22^. Previously many studies have done to determine the antioxidant activity and TPC of different parts of *Ficus auriculata* plant. However, to the best of our knowledge, there are very limited studies on extraction of phenolic and bioactive compounds from fruits extract of *F. auriculata*, in particular no specific study on optimized extraction process.

Therefore, the purpose of this study was to optimize the extraction parameters to extract maximize bioactive and phenolic compounds from the fruits of *F. auriculata* using UAE and RSM. Finally, the phenolic profile of the most active extract was comprehensively studied by liquid chromatography (LC) coupled to mass spectrometry (MS) via electrospray ionization (ESI).

## Materials and methods

### Chemicals and reagents

1,1-Diphenyl-2-picrylhydrazyl (DPPH) and 2,2ʹ-azinobis-(3-ethylbenzothiazoline-6-sulfonic acid) (ABTS) were purchased from Sigma-Aldrich (St. Louis, MO, USA). Folin-Ciocalteu reagent was purchased from *Merck*, Germany. Potassium persulfate, 99.9% pure ethanol, monohydrate gallic acid and anhydrous sodium carbonate were purchased from Friendemann Schmidt (FS) Chemicals, Australia. All the chemicals which were used in this study were in analytical grade. The 18 mΩ deionised water was used to prepare standard materials and extraction.

### Sample preparation

**Figure 1:**
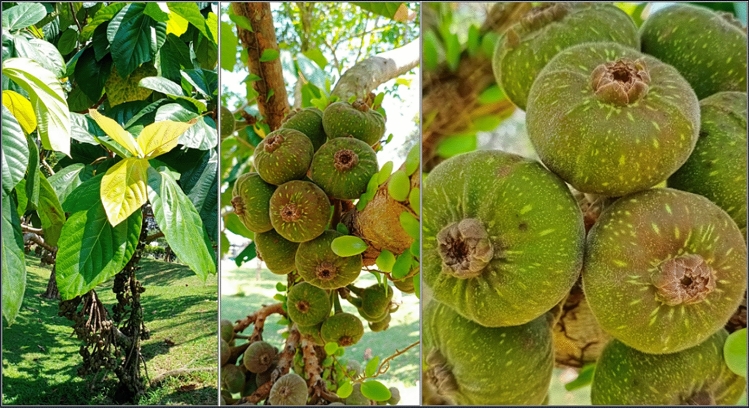
Fresh fruits of *Ficus auriculata* collected from UKM campus.

The fresh fruit samples of *F. auriculata* were picked up from the main campus of Universiti Kebangsaan Malaysia, 43600 Bangi, Selangor, Malaysia (Fig. 1) which was cultivated and maintained by the infrastructure and management, UKM. The permission has been taken to collect the fruit sample from the proper authority (Pengarah Prasarana-UKM) and the collection of fruit sample was done by following the institutional, national and international guidelines and legislation. The plant identity was kindly confirmed by Engineer Mohamad Ruzi Bin Abdul Rahman, Herbarium University Kebangsaan Malaysia (HUKM), Faculty of Science and Technology, Universiti Kebangsaan Malaysia. A voucher specimen (Voucher number: ID002/2021) was deposited at the Herbarium University Kebangsaan Malaysia (HUKM), Faculty of Science and Technology, Universiti Kebangsaan Malaysia. The fruits were cleaned properly with distilled water and then dried at 45–50 °C with the help of Septree Food Dehydrator. Finally, all the fruits were powdered using a special grinder (XY-2200B, Shenzhen Yason General Machinery Co., Ltd, Guangdong, China) and stored in an airtight container.

### Extraction procedures

**Figure 2:**
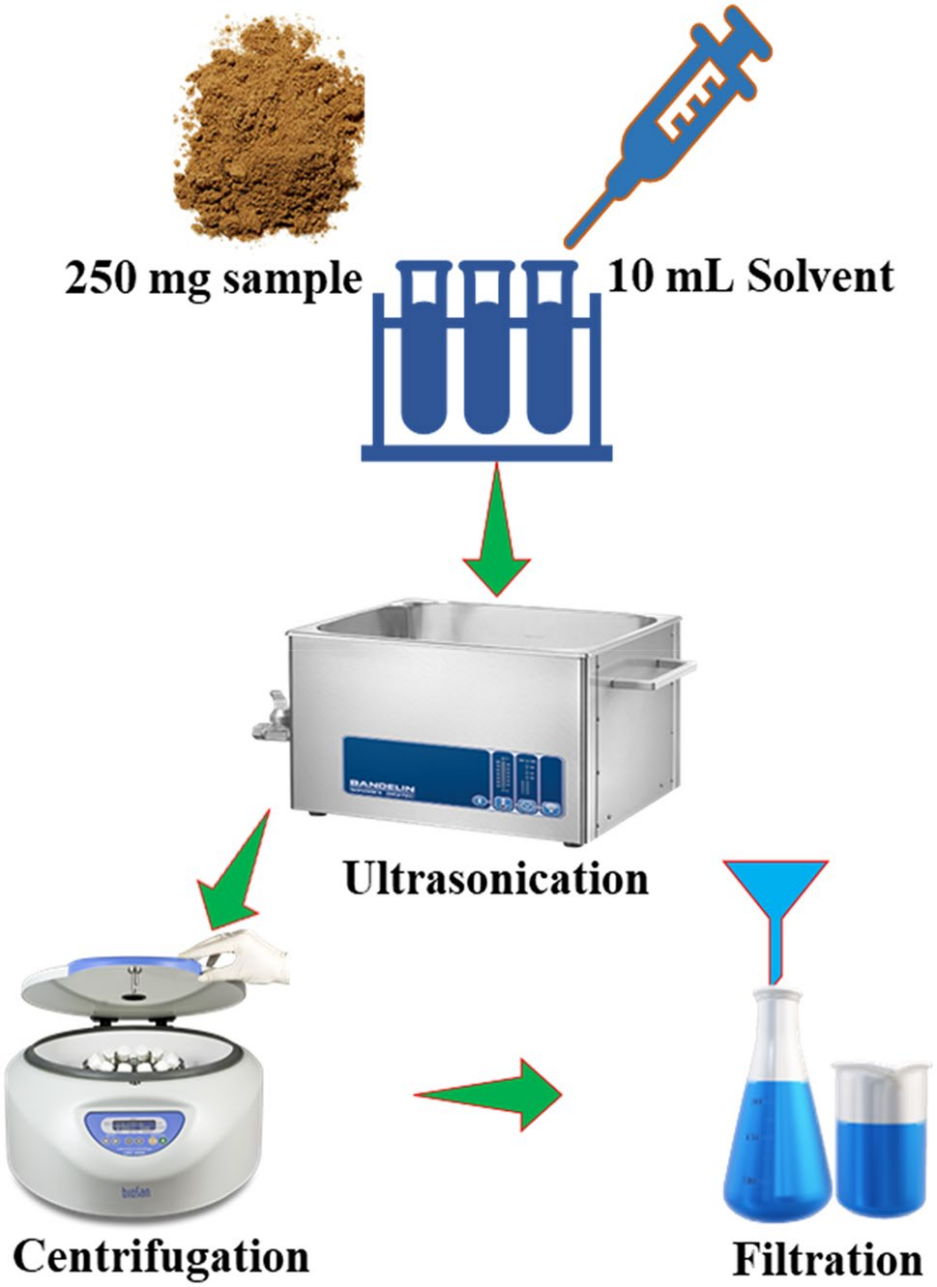
A schematic diagram for extraction process.

The extraction of the fruits of *F. auriculata* was executed in Thermo-line ultrasonic bath (220 V and 40 kHz) at 35 °C. Two hundred fifty mg of dried and ground powdered sample was transferred into a capped long test-tube (50 mL) and 10 mL of solvent was poured in the sample. Then, the mixture was placed in the ultrasonic bath for sonication. Following extraction, the suspension samples were centrifuged for 15 min at 4000 rpm. Finally, the supernatant liquids were filtered, and the extract thus obtained used directly for the determination of required properties. Figure 2 shows the extraction process of antioxidant active compounds from *F. auriculata* fruits.

### Evaluation of antioxidant characteristics

#### DPPH assay

The DPPH radical scavenging activity of fruits extract of *F. auriculata* were measured using previously reported method with some modification^23^. In brief, 0.1 mM of fresh DPPH was prepared with 70% of aqueous ethanol as the control solution. The 100 μL of different standard Trolox solution (positive control) and the sample were added to 3.9 mL DPPH solution (0.1 mM). Then, the control, and sample absorbance were recorded at 520 nm after incubated 30 min at dark condition and room temperature. The DPPH scavenging activity (percentage of inhibition) was calculated by using the equation below:

1 $${\text{Antioxidant capacity }}\left( {\% {\text{ inhibition}}} \right) \, = \, \left[ {\left( {{\text{A}}_{{\text{C}}} {-}{\text{ A}}_{{\text{S}}} } \right) \, /{\text{ A}}_{{\text{S}}} } \right] \, \times {1}00$$

where A_C_ is the absorbance of a control solution, A_S_ is the absorbance of standard or sample solution. Each sample and standard were measured in three replications. The absorbance was measured with 756 PC UV–Visible spectrophotometer.

#### ABTS^+^ assay

The ABTS radical scavenging assay was calculated based on the method described by Gorinstein et al.^24^ with little modifications. At first the 7 mM ABTS solution using water was prepared and mixed with 2.45 mM potassium persulfate (K_2_S_2_O_8_) solution with same ratio to get the free radical solution^25^. In dark condition at room temperature, the mixture was stored for 12–16 h. To carry out each bioassay, the fresh working solution was then made by diluting 1 mL ABTS radical solution with the amount of ethanol needed to achieve an absorbance of 0.700 ± 0.02 units at the wavelength of 745 nm. After that, 100 μL of different standard Trolox solution and extracts sample was added to 3.9 mL of an ABTS^+^ solution and incubated 6 min at room temperature. Finally, the control and sample absorbance were instantly assessed at 745 nm. Here, Trolox is the positive control and 70% of aqueous ethanol is used as blank. Finally the Eq. (1) was used to calculate the inhibition percentage. The equipment used was described before.

### Total phenolic content (TPC) assessment

The TPC of fruits of *F. auriculata* was assessed using Folin-Ciocalteu (FC) reagent with a little modification^26^. Prior to use the FC reagent were diluted at 20 times. Then the 100 μL of gallic acid or extract samples were properly added with 3.4 mL of FC reagent and kept for 7 min. A 500 µL of Na_2_CO_3_ (20%) was then added to the reaction mixture and incubated at room temperature in a dark place for 2 h. The absorbance was finally determined at 760 nm from a standard gallic acid curve of 31.25 µg/mL to 1000 µg/mL. The outcomes of the TPC were presented as mg gallic acid equivalent (GAE)/g dry fruits (DF). Each experiment was done as triplicate. The equipment used was as for previous assays.

### Experimental design

RSM and CCD were used to optimise the three independent variables viz. solvent concentration (X_1_, %, v/v); extraction temperature (X_2_, °C) and sonication time (X_3_, min) at five different levels with responses of two dependent variables such as antioxidant activity (DPPH assay) and TPC (Table 1). The design comprising of 20 experimental runs involving 8 factorial points, 6 axial points, and 6 centre points*. *The second-order polynomial model in the response surface analysis is demonstrated using the Eq. (2):

**Table 1:** Control variables, their coded values and actual values included in optimisation.

Control variables	Units	Symbol	Coded levels				
			− 1.68	− 1	0	+ 1	+ 1.68
Ethanol concentration	%, v/v	X_1_	7.95	25	50	75	92.05
Temperature	°C	X_2_	14.77	25	40	55	65.23
Sonication time	min	X_3_	3.18	10	20	30	36.82

2 $$\mathrm{Y}={\mathrm{B}}_{0}+\sum_{\mathrm{i}=1}^{\mathrm{n}}{\mathrm{B}}_{\mathrm{i}}{\mathrm{X}}_{\mathrm{i}}+ \sum_{\mathrm{i}<\mathrm{j}}^{\mathrm{n}}{\mathrm{B}}_{\mathrm{ij}}{\mathrm{X}}_{\mathrm{i}}{\mathrm{X}}_{\mathrm{j}}+\sum_{\mathrm{j}=1}^{\mathrm{n}}{\mathrm{B}}_{\mathrm{jj}}{\mathrm{X}}_{\mathrm{j}}^{2}$$

where Y is the response function of the independent variables; B_0_ is a constant, B_i_ is the linear coefficient, B_ij_ is the second-order interaction, and B_jj_ is the quadratic coefficients. The variable, X_i_ is the non-coded independent variables. Here, three independent variables were used and hence n equal to 3. Thus, Eq. (2) is expressed with Eq. (3):

3 $$\begin{aligned} {\text{Y }} & = {\text{ B}}_{0} + {\text{ B}}_{{1}} {\text{X}}_{{1}} + {\text{ B}}_{{2}} {\text{X}}_{{2}} + {\text{ B}}_{{3}} {\text{X}}_{{3}} + {\text{ B}}_{{{12}}} {\text{X}}_{{1}} {\text{X}}_{{2}} + {\text{ B}}_{{{13}}} {\text{X}}_{{1}} {\text{X}}_{{3}} \hfill \\ & \quad + {\text{ B}}_{{{23}}} {\text{X}}_{{2}} {\text{X}}_{{3}} + {\text{ B}}_{{{11}}} {\text{X}}_{{1}}{^{{2}}} + {\text{ B}}_{{{22}}} {\text{X}}_{{2}}{^{{2}}} + {\text{ B}}_{{{33}}} {\text{X}}_{{3}}{^{{2}}} \hfill \\ \end{aligned}$$

where Y represents the predicted response (antioxidant activity and TPC), and X_1_, X_2_ and X_3_ are independent variables. B_0_ is a constant and B_1_, B_2_ and B_3_ are linear coefficients. B_12_, B_13_ and B_23_ are cross coefficients and B_11_, B_22_ and B_33_ are quadratic coefficients.

### Statistical analysis

Analysis of variance (ANOVA) was used to verify the statistical validity of the response surface quadratic model coefficients and the Design-Expert 6.0.6 (Stat-Ease, Inc., USA) was used to conduct the data analysis. The regression coefficient (*R*^2^) along with the *F*-test, was assessed to test the fit of the polynomial model. The statistical significances for different terms in the polynomial model were evaluated by the estimation of *F*-value with different probability (*P*) range such as 0.001, 0.01 or 0.05. *P* values less than 0.05 and 0.01 indicate that the value is statistically significant and very significant. The % of DPPH inhibition and GAE curve was done using Microsoft Excel 16 (Microsoft Inc., Redmond, USA).

### Determination of bioactive compounds via LC–ESI–MS studies

The bioactive phenolic compounds were profiled by LC–MS using the mass analyzer Bruker micrOTOF-Q, Bruker, Germany. A reverse phase C18 column (Phenomenex 250 mm, 5 μm particle size) was used. The eluting system consisted of water acidified with 0.1% formic acid and (1:1, v/v) acetonitrile/methanol acidified with 0.1% formic acid as solvent A and B respectively. The 0.45 μm membrane disk filter was used to filter the mobile phase and degassed by sonication before injection. The parameters which were used to the Elution process are as follows: 5% B, 0–5 min; 5%–10% B, 5–10 min; 10%–50% B, 10–55 min; 50%–95% B, 55–65 min; 5% B, 65–70 min. The 20 μL of solvent was injected with 0.4 mL/min flow rate. The analytical parameters with negative ion mode were performed as follows: source temperature 150 °C, desolvation temperature 350 °C, cone voltage 50 eV, capillary voltage 3 kV, cone gas flow 50 L/h, desolvation gas flow 600 L/h. The ion mass spectra were acquired between *m/z* 50–1000 and the peaks data were processed using the Bruker Daltonics Data Analysis 3.4 software. By comparing with the retention time of spectra and reported mass spectrum data with the literature on genus Ficus and family Moraceae, the bioactive compounds were identified.

## Results and discussion

### Impact of solvent on extraction process

**Figure 3:**
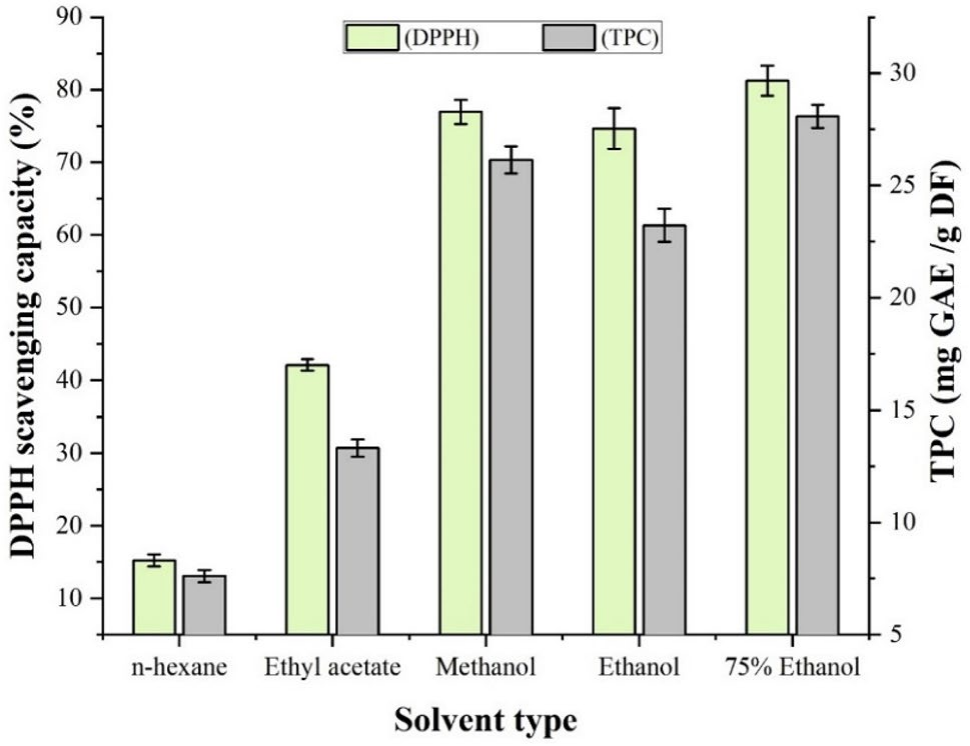
Effect of solvent on the antioxidant activity and TPC of fruits of *Ficus auriculata.* Data are presented as mean ± SD from triplicate experiments.

Before using RSM, the impact of solvent type and solvent to solid ratio were studied. Solvent selection is an important tool for the extraction of plant metabolites. Generally, two polar solvents such as methanol (high polarity) and ethanol (medium polarity), are used for the extraction processes when focusing on phenolic compounds. For the extraction purposes, US Food and Drug Administration (UFDA) recommended environment friend and food-grade non-toxic organic solvents and pure methanol is more toxic than the pure ethanol^27^. In the present study, several of these solvents were used alone or in combination with water. Our results suggested that the efficiency of methanol was higher than single solvent ethanol, ethyl acetate and *n*-hexane, but lower than the aqueous ethanol (75%) to extract phenolic antioxidant compounds from the fruits of *F. auriculata* as per the conditions of 10:0.250 (mL/g) solvent to solid ratio, 40 °C temperature and 30 min extraction time (Fig. 3). From our study, the extraction ability of the bioactive phenolic compounds depends on the polarity of the solvent. In this study four solvents were chosen based on the polarity index with different dielectric constant (ε). Methanol is highly polar solvent where ethanol is medium polar and ethyl acetate is low polar solvent. According to the Fig. 3, n-hexane showed very low activity as it is a non-polar solvent with very low dielectric constant (ε = 1.88). Therefore, due to the low toxicity and better extraction ability of aqueous ethanol, it was chosen as the master solvent for each of the next experimental runs for the determination of antioxidant activity and TPC from the fruits of *F. auriculata*. This agreed with several studies that also found that the combination of water with pure solvent is more effective than solvent alone for extracting phenolic antioxidant compounds^28,29^. So, the aqueous ethanol was the best solvent to extract polyphenols and the addition of water increased the polarity of the ethanol and the extraction potential in this case.

**Figure 4:**
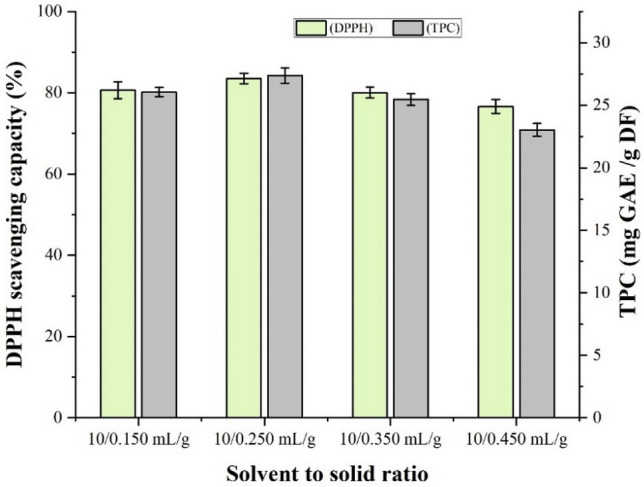
Effect of solvent to solid ratio on the antioxidant and TPC of fruits of *Ficus auriculata* extracted with 75% ethanol. Data are presented as mean ± SD from triplicate experiments.

The influence of solvent to solid ratio on the extraction process from the fruits extract of *F. auriculata* was also studied with four ratio: 10/0.150, 10/0.250, 10/0.350 and 10/0.450 mL/g, over 75% of solvent, 30 min reaction time and 40 °C temperature. Figure 4 presents the outcomes. The antioxidant activities and TPC increased with the increased amount of solid material in a fixed amount of solvent (10 mL), and it increased up to 0.250 mg of solid. After that, the trend followed a declined efficiency. This is because, the speed of mass transfer depends on the ratio of solvent to solid and increasing ratio enable the distribution of antioxidants into the extraction solvent till maximize the mass transfer. Therefore, the ratio of 10/0.250 (mL/g) was chosen for each of the next experimental runs and to minimize the solvent requirement*.*

### Fitting the RSM models

The results (antioxidant activity and TPC values) of the CCD design are shown in Table 2. Moreover, the response surface quadratic model was used to evaluate the extraction process to maximize the inhibition of DPPH and obtain the highest TPC from the fruit extracts of *F. auriculata*. Ethanol concentration (X1), temperature (X2) and time of extraction (X3), were used as the independent variables which also commented before. The regression coefficient (R^2^) was checked to measure the degree of fitness^30^. When R^2^ approaches unity, the model can significantly fit well with the predicted values^31^. The R^2^ value and ANOVA results of the response surface quadratic models for *F. auriculata* fruits extracts are compiled in Tables 3 and 4, respectively. In the present study, *R*^2^ values for antioxidant activity and TPCwere 0.96, for the quadratic model as well as 0.98 and 0.99 respectively for cubic model, but the design suggested quadratic model and aliased cubic model. The high values of R^2^ indicate that there is a good relationship between the predicted and experimental values for the models. The degree of precision of a model also can be checked by the coefficient of variations (C.V.). A high value of C.V. indicates the lower reliability of the experiment^32^. In this study, the C.V. values were 1.17% and 7.47% for antioxidant activity and TPC, respectively, which were low and indicates the executed experiments are highly reliable.

**Table 2:** Experimental design using RSM with CCD for the antioxidant activity (% of DPPH) and total phenolic content (TPC).

Run	Ethanol conc. (%)	Temp (°C)	Time (min)	Antioxidant activity (% of DPPH Inhibition)	TPC (mg GAE/g DF)
	X_1_	X_2_	X_3_	Experimental	Experimental
1	50	40	20	83.48	33.14
2	7.95	40	20	77.23	13.21
3	75	55	30	75.61	25.57
4	75	55	10	77.77	19.03
5	75	25	30	78.93	23.06
6	92.05	40	20	81.87	17.89
7	25	55	30	76.64	19.59
8	50	40	20	83.73	33.18
9	50	14.77	20	81.61	30.65
10	25	25	30	77.41	16.35
11	50	40	20	83.83	33.25
12	25	55	10	77.41	18.78
13	25	25	10	78.28	17.40
14	50	40	3.18	75.87	13.85
15	75	25	10	79.74	18.68
16	50	40	36.82	74.27	25.80
17	50	40	20	84.54	33.41
18	50	65.23	20	81.05	27.07
19	50	40	20	84.92	34.03
20	50	40	20	84.70	33.92

**Table 3:** Adequacy of the model tested for the responses.

Source	Antioxidant activity (% of DPPH inhibition)					Total phenolic content (mg GAE/ g DF)				
	Std. dev.	R^2^	R^2^_Adj_	R^2^_Pre_	C.V.	Std. dev.	R^2^	R^2^_Adj_	R^2^_Pre_	C.V.
Linear	3.61	0.07	− 0.10	− 0.35		7.71	0.09	− 0.06	− 0.31	
2FI	3.99	0.07	− 0.34	− 1.46		8.47	0.11	− 0.29	− 1.31	
Quadratic	0.93	0.96	0.92	0.75	1.17	1.82	0.96	0.94	0.76	7.47
Cubic	0.69	0.98	0.95	− 0.10		0.72	0.99	0.99	0.49

**Table 4:** Analysis of variance (ANOVA) for response surface quadratic model.

Source	Antioxidant activity						Total phenolic content			
	Sum of squares	DF	Mean square	*F* Value	*p* value	Sum of squares	DF	Mean square	*F* Value	*P* Value
Model	220.05	9	24.45	29.46	< 0.0001	1023.42	9	113.71	34.23	< 0.0001
X_1_	7.51	1	7.50	9.04	0.0132	35.75	1	35.74	10.76	0.0083
X_2_	4.53	1	4.53	5.46	0.0416	0.16	1	0.15	0.04	0.8324
X_3_	3.89	1	3.88	4.68	0.0557	69.35	1	69.35	20.88	0.0010
X_1_^2^	46.24	1	46.24	55.72	< 0.0001	613.87	1	613.87	184.8	< 0.0001
X_2_^2^	19.46	1	19.45	23.45	0.0007	47.70	1	47.70	14.36	0.0035
X_3_^2^	164.07	1	164.07	197.73	< 0.0001	362.35	1	362.35	109.1	< 0.0001
X_1_X_2_	1.67	1	1.67	2.01	0.1861	0.38	1	0.38	0.11	0.7419
X_1_X_3_	0.22	1	0.22	0.27	0.6139	15.57	1	15.56	4.68	0.0556
X_2_X_3_	0.20	1	0.19	0.23	0.6373	2.01	1	2.01	0.60	0.4545
Residual	8.30	10	0.83			33.21	10	3.32		
Lack of fit	6.99	5	1.39	5.33	0.0449	32.45	5	6.49	42.71	0.0004
Pure error	1.31	5	0.26			0.76	5	0.15		
Cor total	228.35	19				1056.64	19			

**Figure 5:**
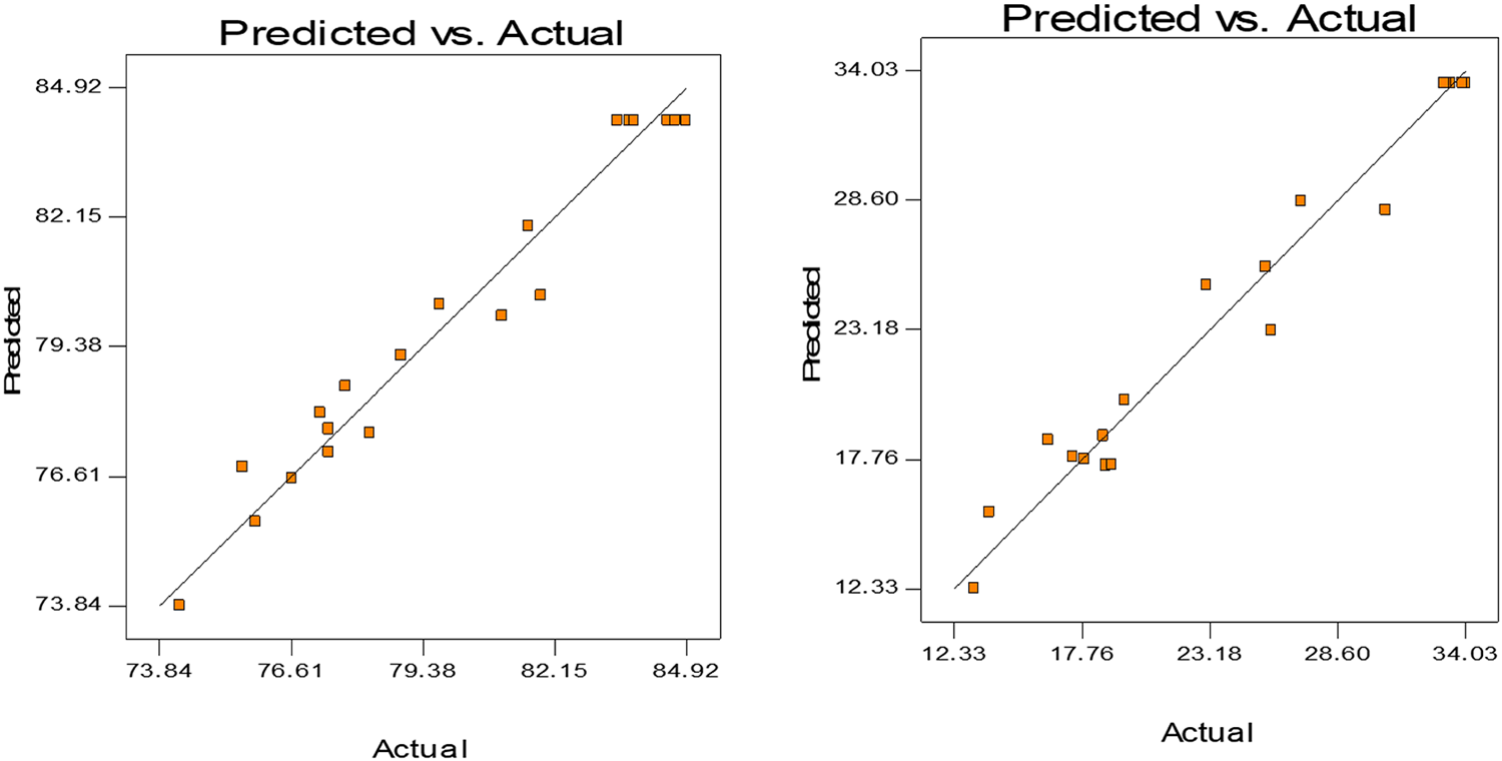
Predicted vs actual values curve for antioxidant activity and TPC of *F. auriculata* fruit extract.

The probability factor (*P*-value) is another important value to evaluate the significance of independent variables. A lower *P*-value is highly recommended for significance^32^. In the present study, the model was significant due to the value of *P* was less than 0.05. According to Table 4, two linear coefficients such as X_1_ and X_2_ and three quadratic term coefficients such as X_1_^2^, X_2_^2^ and X_3_^2^ were significant (*P* < 0.05) for the response of antioxidant activities. In contrast, two linear coefficients (X_1_ and X_3_) and all the quadratic term coefficients (X_1_^2^, X_2_^2^ and X_3_^2^) were significant for the response of TPC. The other terms of coefficients were insignificant due to the *P*-value was > 0.05. Furthermore, the model *F*-value for antioxidant activity and TPC were 29.46 and 34.23, respectively (Table 4). The high *F*-values further confirmed the models significant within the studied range of process conditions. Moreover, the lack of fit for this model also significant (*P*-value < 0.05). Therefore, all the results proved that the model fitness was adequate and both models were fully applicable. Figure 5 represents the Predicted vs Actual values for Antioxidant activity and TPC. The perfect fit line Predicted = Actual values with a high degree of correlation with best fit line equation y = mx + c indicates the best accuracy of the current model.

### Impact of extraction parameters on DPPH assay

**Figure 6:**
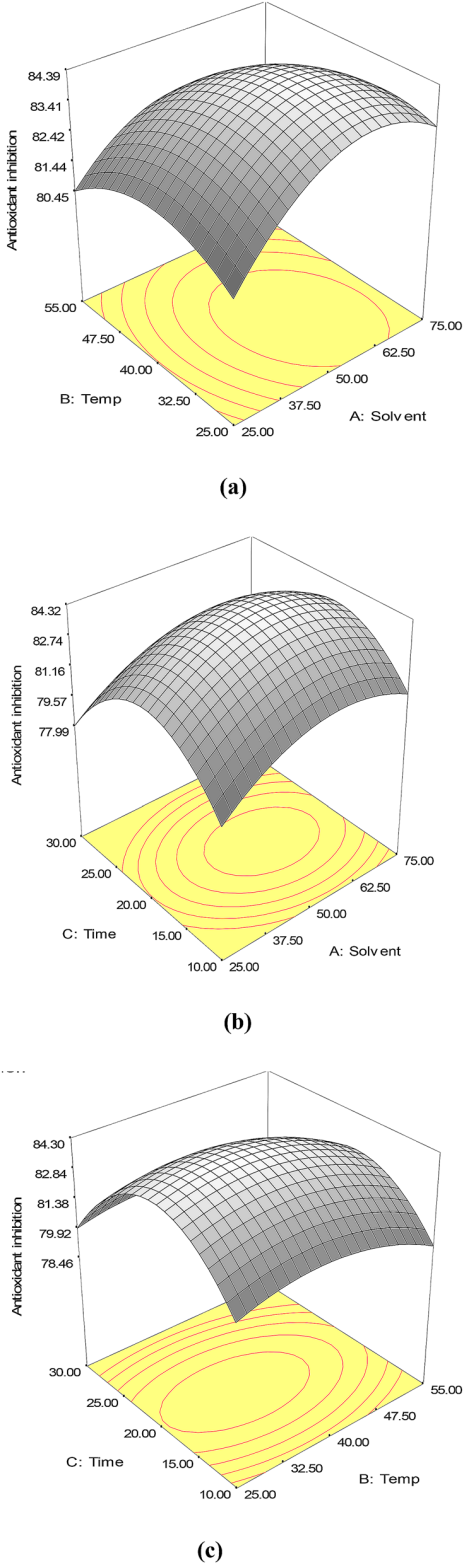
Response surface plots showing the effects of extraction parameters on the DPPH of the extracts from fruits of *F. auriculata*. (**a**) The constant ultrasonic time (20 min), (**b**) the constant temperature (40 °C), and (**c**) the constant ethanol concentration (50%).

The effects of solvent, extraction temperature and sonication time on the DPPH assay of fruit extracts of *F. auriculata* as well as their interactions are shown in Table 2 and Fig. 6. Equation (4) displays the correlation between independent variables for the DPPH radical scavenging activity of *F. auriculata* fruits extracts.

4 $$\begin{aligned} {\text{Y}}_{{1}} \left( {\% {\text{ of DPPH}}} \right) & = 84.21 + 0.74{\text{X}}_{{1}} - 0.57{\text{X}}_{{2}} - 0.53{\text{X}}_{{3}} - 1.76 {\text{X}}_{{1}}{^{{2}}} \hfill \\ & \quad - 1.14 {\text{X}}_{{2}}{^{{2}}} - 3.35 {\text{X}}_{{3}}{^{{2}}} - 0.45 {\text{X}}_{{1}} {\text{X}}_{{2}} - 0.17 {\text{X}}_{{1}} {\text{X}}_{{3}} - 0.15 {\text{X}}_{{2}} {\text{X}}_{{3}} \hfill \\ \end{aligned}$$

where Y_1_ represents the DPPH radical scavenging activity in fruit extracts of *F. auriculata*. X_1_, X_2_, and X_3_ represents the solvent concentration (%), temperature (°C) and time (min), respectively.

The DPPH assay was selected since it is a broadly used and reliable antioxidant determination method compared to other assays^33^. In this process, DPPH solution reduced to non-radical DPPH-H in the presence of hydrogen-donating antioxidants. The antioxidant compound containing crude fruit extract of *F. auriculata* fruits reduced the stable purple colour to yellow-coloured diphenylpicryl-hydrazine. The experimental values of DPPH assay with various extraction conditions are shown in Table 2. The variables studied here, the concentration of ethanol, sonication temperature and sonication time, showed the effects on the antioxidant activity of fruit extract of this plant. At constant sonication time (20 min), the ethanol concentration and temperature effect on DPPH inhibition of *F. auriculata* fruit extract seemed as light-saddled shapes (Fig. 6a). The ethanol concentration (*P* < 0.0001) and the temperature were the main significant extraction parameters for antioxidant activity. The effects of sonication time were not statistically significant (*P* > 0.05), but their quadratic terms were significant as commented before. The DPPH inhibition increases with the growth of ethanol concentration from 7.95 to 52.50%, and thereafter it followed the declining trend at the higher solvent concentration of 92.05%.

The similar trend also found for ultrasound irradiation time for this study (Fig. 6b). The DPPH radical scavenging activities increased from 3 to 22 min and followed by a decreasing trend at longer ultrasound irradiation time. Maximum 84.03% of inhibition was obtained at 22 min. When the ethanol concentration and sonication time were kept constant, the antioxidant activity of the extracts enlarged to a value with the temperature and then started to decrease (Fig. 6c). These studies evidently exhibit that the change of ethanol concentration and temperature, change the activity of DPPH positively in the medium region, and thereafter follow the negative trend for any range of extraction time.

Concerning other studies, similar DPPH radical scavenging antioxidant response plots were also reported by Shahinuzzaman et al. for *F. carica* latex^34^, Yang et al*.* earlier for longan fruit polysaccharides^33^, Ilaiyaraja et al*.* for fruit extract of *Feronia limonia*^35^ and Liyana-Parthirana and Shahidi for wheat extracts^17^. In ultrasound assisted extraction, the DPPH radical scavenging activities of the fruit extract of *F. auriculata* were higher than those of Shirzad and co-worker reported leaves of *Olea europaea* (78.98%)^36^, Li et al*.* reported the antioxidant activity of leaves extract of *P. frutescens* (73.66%)^21^; Ilaiyaraja and co-workers reported fruit extract of *F. limonia* (83.8%)^35^, Tabaraki and Nateghi reported rice bran (52.83%)^18^. Previously, the antioxidant activity with DPPH assay of *Actinidia chinensis* fruit seed extract was studied to optimize the extraction parameters. Using the optimum extraction conditions, it obtained 63.25% antioxidant activity with DPPH assay^37^. However, the maximum antioxidant activity value obtained for *F. auriculata* was lower than that reported for olive leaves (95.56%)^38^.

### Impact of extraction parameters on TPC

The effects of the extraction parameters, on the TPC of fruit extracts of *F. auriculata* under UAE is presents in Table 2. The effect of ethanol concentration and sonication time was decidedly significant as well as the effects of temperature was not statistically significant on the extraction of phenolic compounds. However, multiple regression analysis indicated that the quadratic terms (X_1_^2^, X_2_^2^ and X_3_^2^) were highly significant (*p* < 0.0001) for the extraction of TPC and is revealed in Table 3, as for the antioxidant activity. So, consistent with the experimental values, the model made the second-order polynomial equations to exhibit the correlation between ethanol concentration, temperature, and time for the TPC (Y_2_), and is represented in Eq. (5):

5 $$\begin{aligned} {\text{Y}}_{{2}} ({\text{mg GAE}}/{\text{g DF}}) & = 33.51 + 1.61{\text{X}}_{{1}} + 0.11{\text{X}}_{{2}} + 2.25{\text{X}}_{{3}} - 6.52 {\text{X}}_{{1}}{^{{2}}} \hfill \\ & \quad - 1.82 {\text{X}}_{{2}}{^{{2}}} - 5.01 {\text{X}}_{{3}}{^{{2}}} - 0.22 {\text{X}}_{{1}} {\text{X}}_{{{2} }} + 1.39 {\text{X}}_{{1}} {\text{X}}_{{3}} + \, 0.50{\text{X}}_{{2}} {\text{X}}_{{3}} \hfill \\ \end{aligned}$$

**Figure 7:**
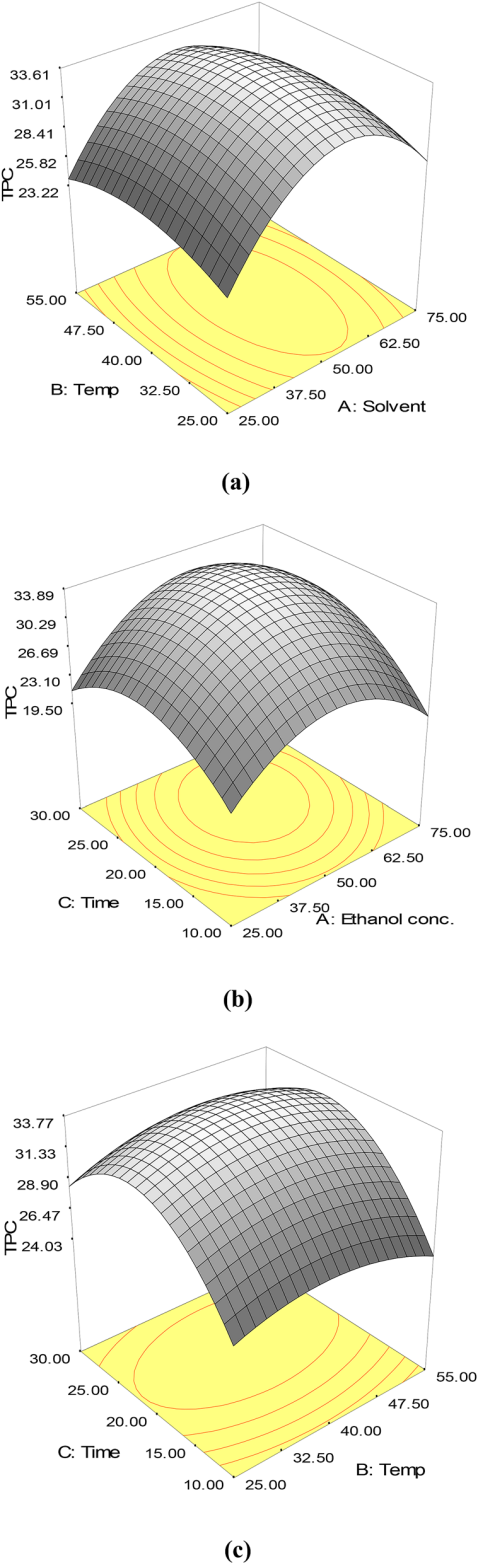
Response surface plots showing effects of ethanol concentration, temperature, and sonication time on total phenolic contents of the extracts from fruits of *F. auriculata*. (**a**) The constant ultrasonic time (20 min), (**b**) the constant extraction temperature (40 °C), and (**c**) the constant ethanol concentration (50%).

A 3D response surface plots were established to obtain the optimum extraction parameters for TPC based on Eq. (5). When sonication time was kept constant (20 min), the effect of solvent and temperature on TPC seemed as a curved shape (Fig. 7a). The TPCs linearly increases with uplifting the ethanol concentration until it reaches a highest limit and then reduced. The highest recovery of phenolics was gained at a solvent concentration between 45 and 55% and temperature between 38 and 43 °C. TPC gradually mounted up and attained a maximum content (~ 33.88 mg TE/g DF) and followed by a sharp decrease afterwards. In this study, the TPC was meaningfully affected by the varying concentration of ethanol and the extraction of phenolic compounds was higher at 52.5% of ethanol thereafter it decreased at the higher concentration of ethanol (75–92.04%). These results are interesting to minimize the global process cost due to the use of ethanol as a solvent.

At constant temperature (40 °C), the relationship of sonication time and concentration of ethanol on TPC is exposed in Fig. 7b. The concentration of ethanol revealed a prominent impact on TPC in a quadratic manner (Table 3). The TPC increases with increasing the ethanol concentration up to 52.5% and additional concentration of ethanol reduced the TPC, probably for the polarity change of the solvent mix. To enhance the yield of phenolic compounds, temperature plays a vital role to soft the plant tissues, increase the solubility and dispersion coefficient of the constituents^35^. In this case, lower recovery of TPC obtained at the higher temperature (65 °C), and 52.5% of ethanol agrees well. The results found from this study are more favourable compared to previous studies which presented equivalent or higher for the fruit extract of *F. limonia*^35^, leaves extract of *P. frutescens*^21^, rice bran^18^, extracts of grape cane^39^, peels extract of *Mangifera pajang*^40^ etc. At constant ethanol concentration, the effect of temperature and sonication time on the yield of TPC is shown in Fig. 7c. TPC of fruit extracts of *F. auriculata* increased sharply with increasing temperature up to 40 °C and thereafter decreased slightly. This phenomenon observed in our study at moderate temperature due to it could soften the plant tissue, weaken the integrity of the cell wall, hydrolyze the bonds between phenol-polysaccharide or phenol–protein and enrich the solubility of phenolics, thus more phenolic compounds would pass to the extraction solvent^41^.

### Validation of the optimal extraction conditions

The optimum operating conditions were performed in DOE software based on each experimental run and combination of the two responses. The goal of this study was to obtain the highest antioxidant activity and yield of total phenolic content from the fruit extracts of *F. auriculata* within the range of extraction parameters. To optimise the extraction parameters of antioxidant activity, an ethanol concentration of 52.5% (v/v), the temperature of 40 °C, and ultrasound irradiation time of 22 min were chosen. The highest TPC also found at the same optimum extraction parameters. These optimum conditions gave the highest response value of 84.03% for DPPH assay and 33.88 mg GAE/g DF for TPC, which was forecasted from the model (Table 5).

**Table 5:** Estimated optimum conditions for DPPH, ABTS and TPC.

Response variables	Optimum UAE condition			Maximum values	
	Ethanol (%)	Temp (°C)	Time (min)	Experimental^a^	Predicted
DPPH (%)	52.5	40	22	85.20 ± 0.96	84.03
ABTS (%)	52.5	40	22	99.12 ± 0.85	–
TPC (mg GAE/ g DF)	52.5	40	22	33.25 ± 0.94	33.88

^a^Data are presented as mean ± SD from triplicate experiments.

The validation of the model was also checked at the predicted conditions. The optimal conditions were also tested by using one more radical scavenging assay, i.e. ABTS assay. The outcomes of the experiments showed the following values: 85.20 ± 0.96% for DPPH, 99.12 ± 0.85% for ABTS and 33.25 ± 0.94 mg GAE/g DF for the TPC, which were reliable with the predictive value. The strong relationship between the predicted and experimental values confirmed that the model is correct and consistent in finding the optimal conditions for antioxidants activity and TPC from the fruit extracts of *F. auriculata.* Therefore, the optimized condition of the proposed protocol can be the easy and time saving way with minimal use of solvent and highest antioxidant activity to extract the bioactive compound from fruits. However, the future trends of this work to keep working with the obtained data is to use other biological assays for antioxidant and TPC as well as the study of sonication power for extraction, harvesting process, season of fruit collection and fertilization.

### Characterization of bioactive compounds at optimized extract using LC–ESI–MS

**Figure 8:**
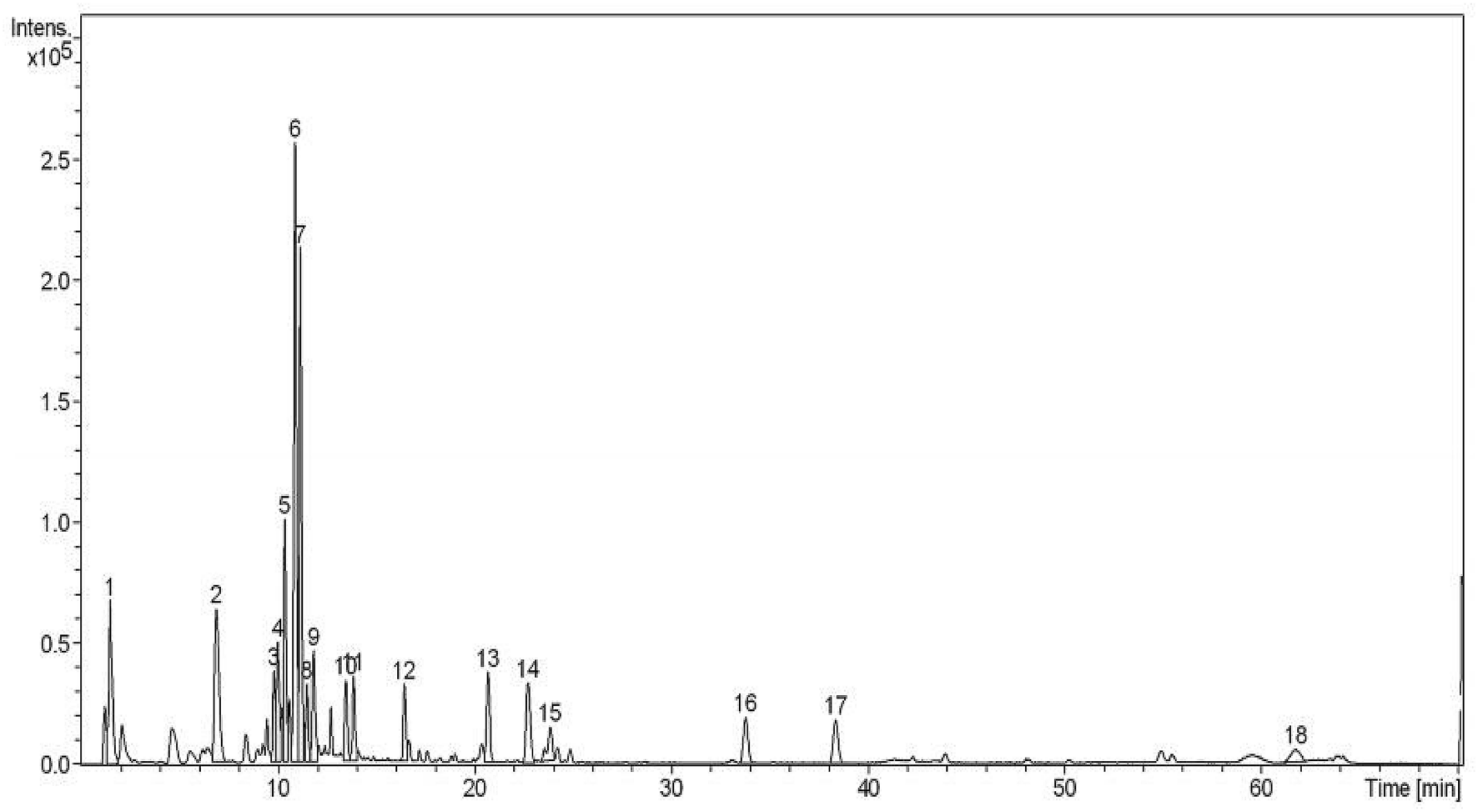
LC–MS fingerprinting analysis of fruits of *F. auriculata* analysed in the negative ionization mode.

The characterization of phenolic compounds was performed by LC–ESI–MS in the negative ionization mode. For that, the most active extract was studied in depth (Fig. 8): fruit of *F. auriculata* extracted through the ultrasonication extraction at the optimised extraction process. The retention time (RT), experimental *m/z* of negative molecular ions ([M−H]^−^), in-source fragments^42^, and the proposed compounds are shown in Table 6. The tentative compounds were compared with the reported literature and databases. A total of 18 bioactive compounds were characterized in *F. auriculata* for first time so far as we know, but few of them were reported in other species. In this way, the preliminary structure of derivatives of caffeoylquinic acid (compounds 2–4), linolenic acid (compound 16) were proposed on the basis of their *m/z* and fragments. For example, the ion *m/z* 353 may indicate the presence of a caffeoylquinic moiety in compounds 2, 3 and 4. The unique hydroxycinnamic acid found in the extract was caffeoylquinic acid (compound 2–4), whose occurrence were previously reported in *F. carica* fruits^43,44^. Flavanols were represented by A-type trimer (compounds 8) (*m/z* 863) as also described Vallejo and co-workers (2012) in *F. carica* fruits^44^. Their fragmentation patterns agreed with previous studies by observing the monomer unit (*m/z* 289), dimer (*m/z* 577), a fragment ion derived from a retro-Diels–Alder fission at *m/z* 425 and its subsequent loss of water (*m/z* 407) depending on the compound^45,46^. Isoflavones consisted of three compounds such as trihydroxy-octadecadienoic acid, trihydroxy octadecanoic acid and hydroxy-octadecatrienoic acid (compounds 10, 11, 13). Most of them have been reported in several *Ficus* species, including *F. carica*, *F. tikoua*, and *F. mucuso*^43,46,47^.

**Table 6:** Tentative identification of chemical constituents of *F. auriculata* fruit by LC-ESI -MS/MS.

Peak no	RT (min)	MW	[M−H]^−^ *m/z*	MS fragments	Identified compounds
1	1.49	342	341	191	Galloylquinic acid
2	6.9	354	353	191	3-*O*-caffeoylquinic acid
3	9.81	354	353	191	4-*O*-caffeoylquinic acid
4	10.00	354	353	191, 179	5-*O*-caffeoylquinic acid
5	10.37	866	865	577, 289	Procyanidin trimer (B-type)
6	10.89	626	625	367, 173	4-*O*-feruloylquinic acid derivatives
7	11.16	610	609	577, 289	Methoxyl-epicatchin dimer
8	11.49	864	863	577, 289	Epicatchin-trimer (A-type)
9	11.83	410	409	277, 173	Unidentified
10	13.47	328	327	211, 171	Trihydroxy-octadecadienoic acid
11	13.85	330	329	211	Trihydroxy octadecanoic acid
12	16.43	488	487	305, 173	Gallocatchin-*O*-hexoside
13	20.68	294	293	275	Hydroxy-octadecatrienoic acid
14	22.70	296	295	277, 171	Xanthone derivatives
15	23.83	472	471	295, 173	3-Methyl epigallocatechin gallate
16	33.75	278	277	173	Linolenic acid
17	38.33	280	279	173	Linoleic acid
18	61.69	572	571	173, 147	Unidentified

These results are highly promising and further studies should be addressed to purify the novel molecules and elucidate their stereochemistry by nuclear magnetic resonance and quantify the compounds using HPLC–DAD-MS, since LC–ESI–MS is limited in this sense.

## Conclusions

UAE is an environmentally friendly, simple, and economical extraction process for the extraction of antioxidants from the fruits of *F. auriculata.* The correlation coefficient of this model was high and suggested that a second-order polynomial model should be used. The highest DPPH and ABTS radical scavenging assay as well as reducing power assays were obtained with the optimized extraction conditions. The predicted and experimental data were almost similar. The profiling of phenolic compounds of the optimized extract by LC–ESI–MS revealed the existence of phenolic acids, flavanols, and isoflavones. This study revealed that the fruits of *F. auriculata* can be a good natural source of antioxidants and phenolic contents. The results of this study supply valuable information to the food and pharmaceutical industries for the extraction of bioactive compounds from the optimized fruit extract. This may also secure the supplementation of bioactive components in a variety of food products aimed at combating oxidative-stress related medical issues through cell metabolism.
